# Forging Ahead in Cardiovascular Disease Management

**DOI:** 10.3390/jcm12175739

**Published:** 2023-09-03

**Authors:** Justyna Domienik-Karlowicz, Michał Ciurzynski

**Affiliations:** Department of Internal Medicine and Cardiology, Medical University of Warsaw, 02-005 Warsaw, Poland

The common threat of cardiovascular diseases (CVDs) constantly holds a dominant position among the leading causes of global mortality.

These diseases are everywhere and, combined with many risks, make studying heart problems both challenging and very important. Just like in other areas of medicine, continuous questions and new ideas help us to make progress. The way we understand and handle heart issues shows how far we have come and what the milestones yet to be achieved are. Against this background, this Special Issue of the *Journal of Clinical Medicine* (JCM), titled “Advances in the Management of Cardiovascular Diseases”, stands out as a leading light with new discoveries and methods that aim to change the way we currently deal with heart problems.

A standout contribution from this issue, by Dobrzycki et al. [1], provides a comprehensive review of state-of-the-art diagnostic and treatment methods of left main coronary artery (LMCA) disease, focusing on percutaneous methods. While there is no one-size-fits-all approach for LMCAD, a team-based approach offers the best care. It is key to note that percutaneous coronary intervention and coronary artery bypass grafting methods complement each other, aiming for the best results in different situations. As we look ahead, more research on LMCA treatments will emerge, but finding the absolute best approach will remain an ongoing journey. In the next article, the authors [2] explore the various effects of acetylsalicylic acid (ASA) following CABG and offer a deeper understanding of how ASA helps beyond just inhibiting platelets. While some of ASA’s effects appear to heighten bleeding risks, this raises questions about whether intensifying ASA treatment post-CABG is advantageous for these patients.

The value of ECG has been re-evaluated by Kubica et al. Their innovative approach seeks to confirm certain initial and post-PCI ECG indicators to predict left ventricular systolic dysfunction following an initial ST-segment elevation heart attack [3].

Further, the exploration of cardiovascular implications of other systemic diseases finds resonance in the insightful study by Pruszczyk et al. [4,5]. Their work on pulmonary embolism and its impact on cardiovascular health provides pivotal insights, reshaping our strategies for this disease management.

In this Special Issue, readers will find invaluable contributions, notably the detailed epidemiological research conducted by Rulkiewicz and her team [6,7]. Their studies shed light on the escalating concerns associated with obesity and smoking, offering a comprehensive exploration into these pressing health challenges.

Navigating through the myriad contributions in this Special Issue, it is palpable that the frontier of CVD management is expansive and ripe for revolutionary breakthroughs. As the Guest Editors, we want to thank our reviewers for their helpful feedback and the JCM team for their hard work. A big thanks to the authors who shared their knowledge in this Special Issue. Together, we hope to make progress in fighting cardiovascular diseases.

## References

[1] Dąbrowski E.J., Kożuch M., Dobrzycki S. (2022). Left Main Coronary Artery Disease—Current Management and Future Perspectives. J. Clin. Med..

[2] Siwik D., Gajewska M., Karoń K., Pluta K., Wondołkowski M., Wilimski R., Szarpak Ł., Filipiak K.J., Gąsecka A. (2021). Pleiotropic Effects of Acetylsalicylic Acid after Coronary Artery Bypass Grafting—Beyond Platelet Inhibition. J. Clin. Med..

[3] Fabiszak T., Kasprzak M., Koziński M., Kubica J. (2021). Assessment of Selected Baseline and Post-PCI Electrocardiographic Parameters as Predictors of Left Ventricular Systolic Dysfunction after a First ST-Segment Elevation Myocardial Infarction. J. Clin. Med..

[4] Dzikowska-Diduch O., Kurnicka K., Lichodziejewska B., Dudzik-Niewiadomska I., Machowski M., Roik M., Wiśniewska M., Siwiec J., Staniszewska I.M., Pruszczyk P. (2022). Electrocardiogram, Echocardiogram and NT-proBNP in Screening for Thromboembolism Pulmonary Hypertension in Patients after Pulmonary Embolism. J. Clin. Med..

[5] Justyna A., Dzikowska-Diduch O., Pacho S., Ciurzyński M., Skowrońska M., Wyzgał-Chojecka A., Piotrowska-Kownacka D., Pruszczyk K., Pucyło S., Sikora A. (2022). Decreased Haemoglobin Level Measured at Admission Predicts Long Term Mortality after the First Episode of Acute Pulmonary Embolism. J. Clin. Med..

[6] Rulkiewicz A., Pilchowska I., Lisik W., Pruszczyk P., Ciurzyński M., Domienik-Karłowicz J. (2022). Prevalence of Obesity and Severe Obesity among Professionally Active Adult Population in Poland and Its Strong Relationship with Cardiovascular Co-Morbidities-POL-O-CARIA 2016–2020 Study. J. Clin. Med..

[7] Rulkiewicz A., Pilchowska I., Lisik W., Pruszczyk P., Domienik-Karłowicz J. (2022). Prevalence of Cigarette Smoking among Professionally Active Adult Population in Poland and Its Strong Relationship with Cardiovascular Co-Morbidities-POL-O-CARIA 2021 Study. J. Clin. Med..

